# Genetic and Antigenic Characterization of Avian Avulavirus Type 6 (AAvV-6) Circulating in Canadian Wild Birds (2005–2017)

**DOI:** 10.3390/v13040543

**Published:** 2021-03-24

**Authors:** Tamiko Hisanaga, Catherine Soos, Nicola Lewis, Oliver Lung, Matthew Suderman, Yohannes Berhane

**Affiliations:** 1 National Centre for Foreign Animal Disease, Canadian Food Inspection Agency, 1015 Arlington, Winnipeg, MB R3E 3M4, Canada; tamiko.hisanaga@canada.ca (T.H.); oliver.lung@canada.ca (O.L.); Matthew.suderman@canada.ca (M.S.); 2 Science and Technology Branch, Environment and Climate Change Canada, Saskatoon, SK S7N 0X4, Canada; Catherine.soos@canada.ca; 3 Department of Pathobiology and Population Sciences, Hawkshead Campus, The Royal Veterinary College, Hawkshead Lane, North Mymms, Hatfield, Hertfordshire AL9 7TA, UK; nilewis@rvc.ac.uk; 4 OIE/FAO International Reference Laboratory for Avian Influenza, Swine Influenza and Newcastle Disease, Animal and Plant Health Agency, Weybridge, Addlestone, Surrey KT15 3NB, UK; 5 Department of Biological Sciences, University of Manitoba, Winnipeg, MB R3T 2N2, Canada; 6 Department of Animal Science, University of Manitoba, Winnipeg, MB R3T 2S2, Canada

**Keywords:** AAvV-6, genome sequence, avian avulavirus (serotype; novel strain)

## Abstract

We describe for the first time the genetic and antigenic characterization of 18 avian avulavirus type-6 viruses (AAvV-6) that were isolated from wild waterfowl in the Americas over the span of 12 years. Only one of the AAvV-6 viruses isolated failed to hemagglutinate chicken red blood cells. We were able to obtain full genome sequences of 16 and 2 fusion gene sequences from the remaining 2 isolates. This is more than double the number of full genome sequences available at the NCBI database. These AAvV-6 viruses phylogenetically grouped into the 2 existing AAvV-6 genotype subgroups indicating the existence of an intercontinental epidemiological link with other AAvV-6 viruses isolated from migratory waterfowl from different Eurasian countries. Antigenic maps made using HI assay data for these isolates showed that the two genetic groups were also antigenically distinct. An isolate representing each genotype was inoculated in specific pathogen free (SPF) chickens, however, no clinical symptoms were observed. A duplex fusion gene based real-time assay for the detection and genotyping of AAvV-6 to genotype 1 and 2 was developed. Using the developed assay, the viral shedding pattern in the infected chickens was examined. The chickens infected with both genotypes were able to shed the virus orally for about a week, however, no significant cloacal shedding was detected in chickens of both groups. Chickens in both groups developed detectable levels of anti-hemagglutinin antibodies 7 days after infection.

## 1. Introduction

Avian avulaviruses are some of the most commonly found viruses infecting a wide variety of domestic and wild birds worldwide [1]. All viruses belonging to the *Avulavirinae* subfamily are pleomorphic, enveloped, single stranded and non-segmented viruses containing a negative sense RNA genome of 10–17 Kb size [1]. The RNA genome of avulaviruses encode six structural proteins (NP, P, M, F, HN, and L) and through RNA editing, two non-structural proteins (V and W) [1,2]. Some AAvV-6 are known to express an additional small hydrophobic (SH) protein from the SH gene located between the F and HN genes [2]. Avulaviruses have two surface spike glycoproteins, the hemagglutinin-neuraminidase (HN) and the fusion (F) protein. The hemagglutinin part of the HN spike protein is responsible for the attachment of virions to the sialic acid containing receptors on the surface of cells and the neuraminidase part has the receptor-destroying activity [3]. A second glycoprotein (F) mediates the fusion of the virion envelope with the cellular plasma membrane and sometimes plays role in syncytia formation [4]. Presence of single or multiple basic amino acids at the fusion protein cleavage site is a key indicator of virulence of the virus [5].

Viruses of the subfamily *Avulavirinae* were previously subdivided into different serogroups based on their cross reactivity in hemagglutination inhibition (HI) and/or neuraminidase inhibition (NI) assays [1]. However, due to problems associated with cross-reactivity among some serotypes of avulaviruses in serologic assays, Miller et al. [6] advocated for new classification guidelines that also includes the use of genomic sequence comparisons in the classification of avulaviruses. The International Committee on Taxonomy of viruses has recently reclassified the family *Paramyxoviridae*, elevating *Avulavirinae* to a fourth subfamily group based upon the phylogenetic analysis of the large polymerase (L) gene [7]. Based on this new classification, there are currently 3 genera within the subfamily of *Avulavirinae*: *Orthoavulavirus*, *Paraavulavirus* and *Metaavlulavirus*. Avian avulavirus 6 (AAvV-6) along with AAvV-2, AAvV-5, AAvV-7, AAvV-8, AAvV-10, AAvV-11, AAvV-14, AAvV-15 and AAvV-20 belong to the genera *Metaavulvirus*.

Most studies have detected these viruses during surveillance studies targeting other viruses such as influenza. These viruses have been isolated from a wide variety of wild birds throughout Europe and Asia [2,8,9,10,11,12,13,14]; but have not been widely reported elsewhere. A serological survey from 100 commercial poultry flocks in the United States found evidence for AAvV-6 antibodies to be present in domestic poultry flocks [15]. Initially, a seroprevalence of 47% was found, however due to the cross-reactivity of high titre AAvV-1, when the titers were adjusted to a 128-threshold titer, a seroprevalence of 21% for AAvV-6 was detected. Although not purported to be as pathogenic, AAvV-6 infections in turkeys have been implicated in causing mild respiratory disease and decreases in egg production [2,9]. The isolation of strain It4524-2 in 2010 [9] provided the first evidence of AAvV-6 having two groups. The length of the viral genome is six nucleotides shorter, antigenically displays up to 8-fold lower HI titres to existing prototype sera and had genetic distance of 29–31% from existing AAvV-6 viruses. An influenza surveillance study in Korea increased the known number of group 2 viruses to eleven, allowing for robust justification of the second group [13]. Finally, there were at least four independent viruses in the second group and they displayed a 44% mean evolutionary distance between the groups, satisfying the validation of a second genotype.

The emergence of A/goose/Guangdong/1/96 (Gs/GD) (H5N1) lineage highly pathogenic avian influenza (HPAI) in Hong Kong in 1997 and the potential for viruses from this lineage to be transported to North America via intercontinental migratory waterfowl, impelled many countries to conduct avian influenza surveillance in wild waterfowl 16]. In response to the emergence of Gs/GD lineage H5N1 viruses coupled with the 2004 outbreak of H7N3 HPAI in Fraser Valley, BC, Canada’s Inter-agency Wild Bird Influenza Survey was initiated in 2005 [16,17]. This promoted the isolation and discovery of many different subtypes of avian influenza viruses and also led to the serendipitous isolation of more AAvVs from wild waterfowl. In the current study, we describe the molecular and biological characterization of AAvV-6 viruses that were isolated from the routine avian influenza surveillance in wild waterfowl in Canada from 2005 to 2017.

## 2. Materials and Methods

### 2.1. Sample Collection and RNA Extraction

Samples collected from live birds as part of Canada’s Inter-agency Wild Bird Influenza Survey were collected in conjunction with ongoing waterfowl banding activities led by Environment and Climate Change Canada (ECCC), provincial wildlife agencies, and United States Fish and Wildlife Service (USFWS). A single cloacal swab was collected in 2005, and from 2006–2017, both a cloacal and oropharyngeal swab were collected and placed into a single vial containing modified Hanks buffer, and stored as described in Parmley et al. [17,18]. During this period thousands of samples were collected from wild waterfowl in different provinces of Canada. Total RNA was extracted from the swab samples as previously descried [16]. Commencing in 2012, total RNA was extracted using the MagMAX-96 viral isolation kit (Ambion, Austin, TX, USA) with the KingFisher processor (Thermo Scientific, Waltham, MA) and initial screening with real-time reverse transcriptase PCR (RT-PCR) for the AI virus matrix gene was modified as described by Weingartl et al. [19]. Avian influenza positive samples were processed further for virus isolation.

All procedures involving experimental animal inoculations and care complied with the Canadian Council of Animal Care guidelines and were approved under AUD C-18–001, issued August 2018. Field sampling was carried out in accordance with the Canadian Council of Animal Care guidelines.

### 2.2. Virus Isolation

Swab samples were processed for virus isolation using 9–10 day old specific pathogen free (SPF) embryonating chicken eggs as described previously [16]. After inoculation, the embryos were checked daily for mortality. Embryos that died within the first 24 hrs following inoculation were discarded. Amnio-allantoic fluids (AAF) collected from embryos that died after 24 hrs were harvested and assayed for hemagglutination activity (HA) using 0.5% (*v*/*v*) suspension of chicken red blood cells (CRBC). In addition, AAF fluids from live embryos at the end of first and second passages (6 and 7 days post inoculation, respectively) were tested for the presence of hemagglutinating agents by HA test.

### 2.3. Hemagglutination Inhibition (HI) Assay

Hemagglutination-inhibition (HI) assays were carried out using standard procedures as described in the WHO manual [20]. AAF that tested positive on the HA assay, were further tested in HI assay using a panel of reference antisera prepared against H1 to H16 subtypes of avian influenza virus and against AAvVs 1 to −9 (excluding AAvV-5). The reciprocal of the highest dilution of serum that completely inhibited hemagglutination was considered the HI titer.

### 2.4. Sequencing

Nucleic acids were extracted using the MagMAX Pathogen RNA/DNA Kit (Ambion) according to the manufacturer’s instructions. cDNA synthesis was then performed using SuperScript IV First Strand Synthesis System and random hexamers. Second strand synthesis was carried out using the NEBNext mRNA Second Strand Synthesis Module (New England Biolabs, Whitby, Canada) and the resulting double-stranded cDNA was purified with Qiagen’s Qiaquick PCR Purification Kit. Samples were quantified on the Qubit 3.0 fluorometer using the dsDNA High Sensitivity kit (Thermofisher Scientific, Mississauga, ON, Canada) and sequencing libraries were generated with the Nextera XT Library Preparation kit (Illumina Inc., San Diego, CA, USA). Sequencing was performed on the Illumina MiSeq platform using a V2 300-cycle (2 × 150 bp reads) cartridge and Microflow cell.

### 2.5. Phylogenetic Analysis

The quality of the sequences used for phylogenetic analysis were assessed using FastQC and trimmed using Trimmomatic (version 0.36) [21]. Raw data sequences were analyzed and separately assembled de novo using DNASTAR’s LaserGene 15 with Seqman NGen software’s default parameters (Madison, WI, USA). Open reading frames were predicted using Genequest. The BLASTn program was used to search for homologous genes or genome sequences. Multiple sequence alignments were performed using MAFFT [22] and imported into MEGA X [23] for phylogenetic analysis of the fusion gene and full AAvV-6 genomes. Goodness-of-fit was measured by the corrected Akaike information criterion.

### 2.6. Development of a Duplex Fusion Gene Based Real-Time RT-PCR for AMPV-6

Real-time RT-PCR primers and probes were designed from a conserved region of the AAvV-6 fusion gene that serve both the simultaneous detection and differentiation of the existing two genotypes of AAvV-6. Briefly, multiple sequence alignment was performed using fusion genes from all AAvV-6 viruses from this study and those that were available at NCBI virus database during this study. Alignment of the fusion genes was performed with MAFFT. Multiple primers and Taqman based hydrolysis probes were designed from a conserved region. After in silico analysis, 2 sets of forward primers, a single reverse primer and two probes were selected (Table 1).

### 2.7. Fusion Real-Time RT-PCR Conditions

Real-time RT-PCR was performed on the Roche LC480 using the AgPath-ID One-step RT-PCR kit (Ambion). Each real-time RT-PCR run contained 25 μL of total reaction volume. The RT-PCR mix contained 1 μL cocktail of 25× primer and probe mix (0.125 µM of each probe, 0.2 µM of each primer), 12.5 μL of 2× buffer, 25× enzyme mix, 1.67 detection enhancer, 0.83 μL of RNase free water and 8 μL of template. The real-time RT-PCR reaction conditions were as follows: 10 min at 45 °C and 10 min at 95 °C, followed by 45 cycles of 10 s at 94 °C, 30 s at 50 °C and 30 s at 72 °C.

### 2.8. Biological Characterization of AAvV-6 Viruses in Chickens

For this purpose, twenty-five 6-week old specific pathogen free (SPF) white Leghorn chickens were obtained from CFIA Fallow-field laboratory, Ottawa. The chickens were split into three different groups: A (10 chickens), B (10 chickens) and C (5 chickens) upon arrival and were floor housed in heated BSL3 animal cubicles at National Center for Foreign Animal Disease (NCFAD). After one week of acclimatization, blood and swabs (cloacal and oral) were collected from chickens of all 3 groups prior to inoculation. Chickens in group A were infected with 10^7.4^ 50% egg infectious dose (EID_50_) AAvV-6 BWTE/SK/109-1732/2016 and group B with 10^7^ EID_50_ AAvV-6 mallard/QC/675/2005. Group C chickens were mock inoculated with PBS. All chicken groups were inoculated using a combination of intraocular and intranasal routes to mimic natural infection. Oral and cloacal swabs were collected from chickens of all 3 groups at 3, 5, 7 and 10 days after inoculation. Blood samples were collected at 0, 7, 14, 21 and 28 days after inoculation. To determine the shedding pattern of chickens inoculated with both viruses, total RNA was extracted from each swab sample as described above and presence of AAvV-6 genomic material was determined using the fusion real-time RT-PCR described above.

### 2.9. Serology

Serum samples collected from all chicken groups at 0, 7, 14, 21 and 28 days post-infection were heat inactivated at 56 °C for half an hour. Presence of anti AAvV-6 anti-hemagglutinin antibodies were determined in hemagglutination inhibition assay as described above using homologous and heterologous virus antigen. The reciprocal of the highest dilution of serum to completely prevent agglutination of chicken RBC by virus was considered the HI titer for that serum sample. Antigenic drift and/or relatedness was inferred by comparisons between the HI titer of homologous and test viruses and by antigenic cartography as described below.

### 2.10. Antigenic Cartography

For antigenic cartography, the cross-hemagglutination inhibition test results of the AAVV-6 viruses and corresponding antisera produced against AAvVs-6 representing each genotype were performed. The cross-hemagglutination test results are summarized in Table 2. The antisera used in the HI assays were generated from AAvV-6 isolates representing each genotype based on phylogenetic tree analysis and the viruses included duck/Hong Kong/18/199/77, BWTE/SK/109-1732/2016 and mallard/QC/675/2005. The generated cross-hemagglutination inhibition data was used to create an antigenic map showing the antigenic relationships among the Canadian AAvVs-6 isolates and reference AAvVs -6 using ac-macs web-based software as described previously [24,25]. The distance between points in an antigenic map best represents the antigenic distance among strains as measured in the HI assay. Strains that are closely related antigenically are closer to each other and those that are not will be more distant in the map.

## 3. Results

### 3.1. Virus Isolation and Characterization

Eighteen swab samples collected between 2005 and 2017 from wild waterfowl that initially tested positive for the presence of influenza A virus genomic material by real-time RT-PCR assay were inoculated into 9-day-old SPF embryonating chicken eggs. The majority of these samples caused embryo mortality, however, one sample (AAvV-6 mallard/QC/610/2005) did not result in embryo mortality. The AAF collected from this sample at the end of 2nd passage tested positive by HA assay. Almost all these isolates were able to hemagglutinate chicken red blood cells with titers ranging from 16 to ≥4096. However, one sample, produced an isolate (AAvV-6 mallard/MB/111-986/2017) that although failed to hemagglutinate chicken RBC. Electron microscopy detected the presence of pleomorphic paramyxovirus-like particles with visible herringbone-like structure. Upon further investigation, the virus was determined to be an AAvV-6 based upon sequencing. Hemagglutination inhibition (HI) assay was carried out using a panel of reference antisera prepared against influenza A viruses of H1 to H16 subtypes and against AAvVs 1 to −9 (excluding AAvV-5). Seventeen out of 18 samples cross reacted with reference AAvV-6 antiserum (AAvV-6 Dk/HongKong/18/199/77) with HI titers ranging from 4 to 128. These isolates did not cross react with any of the reference influenza A virus subtype antisera used in the HI assay. In addition, no significant cross-reactivity was observed to other avian avulavirus serotypes used in the HI test panel. To exclude presence of mixed infection, total RNA was extracted from the AAF of all samples that yielded AAvV-6 viruses and tested for the presence of influenza A virus and AAvV-1 genomic material using corresponding real-time RT-PCR assays. All samples tested negative for the presence of influenza A genomic material, but were positive for the presence of AAvV-6 genomic material. Results of virus isolation are summarized in Table 3.

### 3.2. Molecular and Phylogenetic Characterization

Of the eighteen AAvV-6 viruses isolated in this study, complete genome sequences were obtained for fifteen viruses. The genome for strain AAVV-6 mallard/QC/610/2005 was incomplete, missing the first 14 nucleotides of the 5′ end of the trailer region of the genome. Only the fusion gene was obtained for AAvV-6 American wigeon/NS/664/2005 and AAvV-6 mallard/QC/713/2005. Two strains AAvV-6 BWTE/SK/109-1732/2016 and AAvV-6 mallard/QC/544/2005 had similar genome lengths (16,236 nt) to the prototype AAvV-6 Dk/HongKong/18/199/77 strain. The remaining strains were all six nucleotides shorter (16,230 nt) in genome length, similar to the AAvV -6/red-necked stint/Japan/8KS0813/2008 isolate that forms a second subgroup. The 3′ leader sequence was strongly conserved within the subgroups, with AAvV-6 BWTE/SK/109-1732/2016 differing in only 2 of 55 nucleotides when compared to the AAvV-6 Dk/HongKong/18/199/77 strain, and AAvV-6 mallard/BC/704/2005 differed in 1 of 55 nucleotides when compared to AAvV-6 red-necked stint/Japan/8KS0813/2008. Conversely, the 5′ trailer sequence was less conserved with AAvV-6 BWTE/SK/109-1732/2016 having 4 differences and AAvV-6 mallard/QC/544/2005 differing in 3 nucleotides out of 54 when compared to the HK strain. For the second subgroup, AB139, AB150, ON498, MB111-982 and MB111-986 each had 1 nucleotide of 54 difference and finally BC704 differed in 2 of 54 nucleotides from 8KS0813. The proposed gene start signal sequences were strongly conserved among all strains with the lone exception being AAvV-6 mallard/ON/498/2005 which had the signature of GAGGGAGAAC for M and SH genes. The gene end signal sequences were more variable based along subgroup divisions. The SH gene end signal sequence was variable in terms of length of adenosines; and occurred amongst strains in both genotypes.

**Figure 1:**
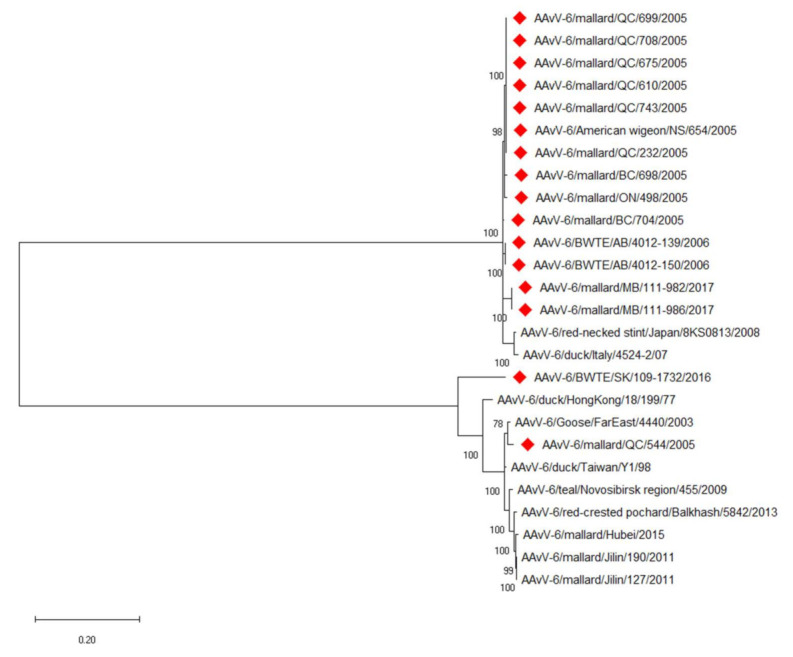
Phylogenetic analysis of the whole genome sequences of AAvV-6 viruses. Viruses described in this study are denoted by red diamonds. The evolutionary history was inferred by using the Maximum Likelihood method based on the General Time Reversible model. The tree with the highest log likelihood (−61096.09) is shown. The percentage of trees in which the associated taxa clustered together is shown next to the branches. Initial tree(s) for the heuristic search were obtained automatically by applying Neighbor-Join and BioNJ algorithms to a matrix of pairwise distances estimated using the Maximum Composite Likelihood (MCL) approach, and then selecting the topology with superior log likelihood value. A discrete Gamma distribution was used to model evolutionary rate differences among sites (5 categories (+*G*, parameter = 0.6440)). The rate variation model allowed for some sites to be evolutionarily invariable ([+*I*], 31.21% sites). The tree is drawn to scale, with branch lengths measured in the number of substitutions per site. The analysis involved 26 nucleotide sequences. There were a total of 16,420 positions in the final dataset. Evolutionary analyses were conducted in MEGA X.

**Figure 2:**
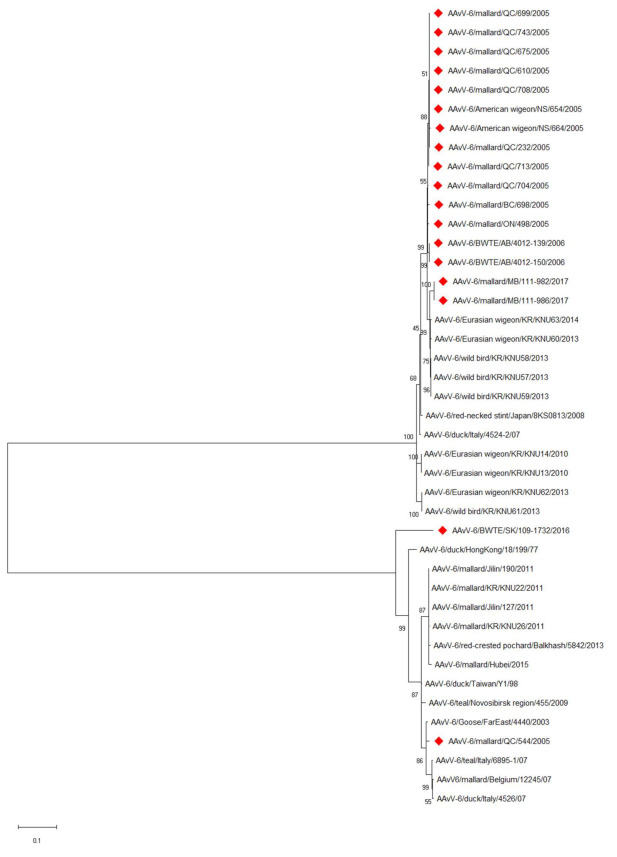
Phylogenetic analysis of the fusion gene sequences of AAvV-6 viruses. Viruses described in this study are denoted by red diamonds. The evolutionary history was inferred by using the Maximum Likelihood method based on the General Time Reversible model. The tree with the highest log likelihood (−6759.79) is shown. The percentage of trees in which the associated taxa clustered together is shown next to the branches. Initial tree(s) for the heuristic search were obtained automatically by applying Neighbor-Join and BioNJ algorithms to a matrix of pairwise distances estimated using the Maximum Composite Likelihood (MCL) approach, and then selecting the topology with superior log likelihood value. A discrete Gamma distribution was used to model evolutionary rate differences among sites (5 categories (+*G*, parameter = 0.5780)). The rate variation model allowed for some sites to be evolutionarily invariable ([+*I*], 31.74% sites). The tree is drawn to scale, with branch lengths measured in the number of substitutions per site. The analysis involved 42 nucleotide sequences. There were a total of 1668 positions in the final dataset. Evolutionary analyses were conducted in MEGA X.

The fusion gene cleavage site sequence for prototype genotype AAvV-6 viruses (PEPR/L) was identical in the two Canadian genotype 1 isolates. The Canadian genotype 2 isolates also shared identical cleavage sites (REPR/L) and also matched previously reported genotype 2 viruses (Table 3). A phylogenetic tree of the full genomes (Figure 1) reinforces the subgrouping of the viruses into genotype 1 and 2 viruses previously reported [9,12,14]. Only two of the viruses sequenced in this study are from genotype 1. The BWTE/SK/109-1732/2016 strain occupies a distinct branch in the genotype 1 cluster indicating the potential existence of another subgenotype. Genetic variation amongst the genotype 2 viruses is lower than that seen in the genotype 1 viruses based upon phylogenetic analysis. The fusion gene sequences of two additional viruses are depicted in Figure 2, a phylogenetic tree of the fusion gene. These additional viruses (AAvV-6 American wigeon/NS/664/2005 and AAvV-6 mallard QC/713/2005) both cluster in the genotype 2 group, along with most of the Canadian AAvV-6 viruses. Two genotype 2 viruses isolated from mallards in 2017 form a branch distinct from those isolated in 2005–2006. The relationships and clustering of the viruses remains similar to what is seen in the full genome phylogenetic analysis. The positioning of BWTE/SK/109-1732/2016 is also similar to the that seen at the genome level.

Evolutionary distances between and within groups were calculated based upon the complete fusion gene sequences using MEGA X. Analyses were conducted using the Maximum Composite Likelihood model (supplementals S1 and S2). Estimates of evolutionary intragroup distances for genotype 1 (*n* = 15) and genotype 2 (*n* = 27) were 5.76% and 2.33%, respectively. The mean inter-populational evolutionary distances between genotypes 1 and 2 was estimated at 91.9%.

When the differences seen amongst the genomes are investigated in further detail, the non-coding regions of AAvV-6 strain BWTE/SK/109-1732/2016 as expected, account for a greater number of differences when compared to other genotype 1 counterparts, however, differences are noted throughout coding regions of the genome as well. Amongst the coding regions, the greatest differentiation is seen in the SH gene, followed by the non-structural genes.

Co-transcriptional RNA editing of the P gene produces two mRNAs that code for accessory viral proteins; V and W. Bioinformatic analysis of the AAvV-6 P genes demonstrate identical N-termini, and variable C-termini sequences. The C-terminus variability was greater in the V proteins than the W proteins. The W and V proteins for the isolates presented in this paper can be found in Supplementals S3 and S4, respectively. The predicted RNA editing sites (UUUUUUCCC) and cis-acting sequences were as previously reported [26]. Little variation is seen amongst the W proteins of genotype 2 viruses, whereas minor variation is seen amongst the genotype 1 viruses, with mallard/QC/544/2005 being most similar to previously published AAvV-6 W proteins. BWTE/SK/109-1732/2016 is quite distinct, with differences throughout all regions save the N-terminus from other genotype 1 viruses. For the genotype 1 viruses isolated in Canada, similar patterns of difference in the V proteins are seen as those in the W proteins.

### 3.3. Development of a Duplex Fusion Gene Based Real-Time RT-PCR for AAvV-6

A duplex fusion gene based real-time assay for the detection and genotyping of AAvV-6 to genotype 1 and 2 was developed. The primers and probes were derived from a conserved region of the fusion gene (nucleotides 252–443 based upon the HK strain) and are described in Table 1. The primers (For-1 and Rev 1-2) and (For-2 and Rev 1-2) generated 191 bp PCR amplicons from viruses corresponding to each genotype; AAvV-6/Dk/HongKong/18/199/77 for genotype 1 and AAvV-6/American wigeon/NS/654/2005 for genotype 2. The amplicons were cloned to into the TOPO-TA pCR4 vector (Invitrogen) to generate controls for the duplex real-time RT-PCR assay. Based upon a plasmid dilution series, the limit of detection was determined to be 8.64 × 10^1^ and 1.69 × 10^2^ copies for genotypes 1 and 2, respectively. When Canadian isolates cultured from SPF embryonated chicken eggs were subjected to the duplex RT-PCR, no cross-reactivity was observed. To ensure assay specificity, RNA from isolates of other avian viruses of importance [AAvV 1-3 and various influenza A viruses (H1 to H16 subtypes)] were tested. None of the RNA from these viruses were detected.

### 3.4. Biological Characterisation AAvV-6 Viruses in SPF Chickens

Leghorn chickens inoculated with AAvV-6/BWTE/SK/109-1732/2016 and AAvV-6/mallard/QC/675/2005 did not exhibit any visible clinical signs after inoculation. Serum collected prior to infection of the chickens were all negative for AAvV-6 antibodies, indicating no prior exposure to the virus. Serum collected at 7 dpi contained detectable levels of anti-hemagglutinin antibodies to the virus in only two of the ten chickens, however, seroconversion had occurred in all chickens by 14 dpi. Homologous HI titers for BWTE/SK/109-1732/2016 infected birds ranged from 32–256 and sampling points at 21 dpi indicate a slight dropping of HI titers by 2-fold, which remained consistent through to 28 dpi. A similar pattern was seen for antibodies developed against AAvV-6/mallard/QC/675/2005 infection, with homologous titers obtained ranging slightly lower, from 32–128. Convalescent antisera from chickens raised against genotype 1 and 2 viruses was assayed against select viruses isolated and described in this study (Table 2). The HI assay showed that the antisera reacted with all seven of the viruses tested. However, the strains can be easily segregated into two categories based upon HI titers that mimic the two genotypes. Typically, the HI titers for viruses of similar genotype based upon phylogenetic analysis is 8 fold greater than for viruses of differing genotypes. For example, the homologous HI titer for mallard/QC/675/2005 chicken #602 antisera is 128, and the titers for other genotype 2 viruses are only 2-fold less at 64. However, the HI titers of the genotype 1 viruses is 8-fold less at 8. For BWTE/SK/109-1732/2016 chicken #485 the difference in titers is less drastic, likely due to the lower homologous titre of the virus. When the prototype duck/Hong Kong/18/199/77 antisera is assayed, less dramatic differences in antigenic variation are seen amongst the two groups. This reference antiserum was produced by boosting chickens a couple of times to reach a higher titre, thus resulting in the observed cross reactivity.

**Figure 3:**
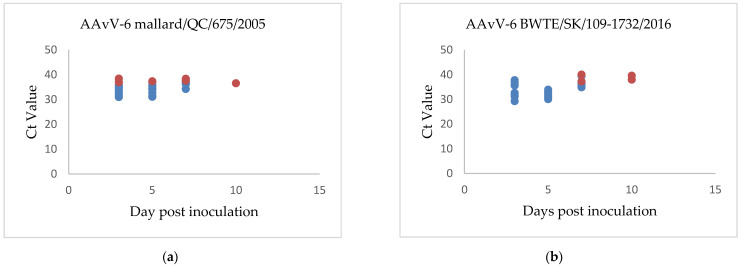
Shedding patterns of chickens inoculated with (**a**) AAvV-6 strain BWTE/SK/109-1732/2016 and (**b**) AAvV-6 strain mallard/QC/675/2005. Oral shedding Ct values are shown in blue, cloacal shedding Ct values are shown in red.

The duplex real-time RT-PCR assay described here was used to determine shedding patterns of the inoculated birds. Swabs collected from chickens of all groups at 0 dpi tested negative for the presence of genomic material of the virus. In chicken groups inoculated with genotype 1 virus BWTE/SK/109-1732/2016, the presence of virus was detected in oropharyngeal swabs of all 10 inoculated chickens at 3 and 5 dpi; waning in detection at 7 dpi, with virus detected in 6 chickens and none by 10 dpi (Figure 3a). Weak cloacal shedding was detected at 7 and 10 dpi in a few chickens. Similarly, AAvV-6/mallard/QC/675/2005, a genotype 2 virus, was detected primarily in oropharyngeal swabs. Similar to the shedding pattern of it is genotype 1 counterpart, virus was detected in all chickens at 3 and 5 dpi swabs, in only seven chickens by 7 dpi and undetectable at 10 dpi (Figure 3b). Low and consistent cloacal shedding was observed in one chicken starting from 3 dpi to 10 dpi and accounts for majority of cloacal shedding observed in group B.

### 3.5. Antigenic Cartography

**Figure 4:**
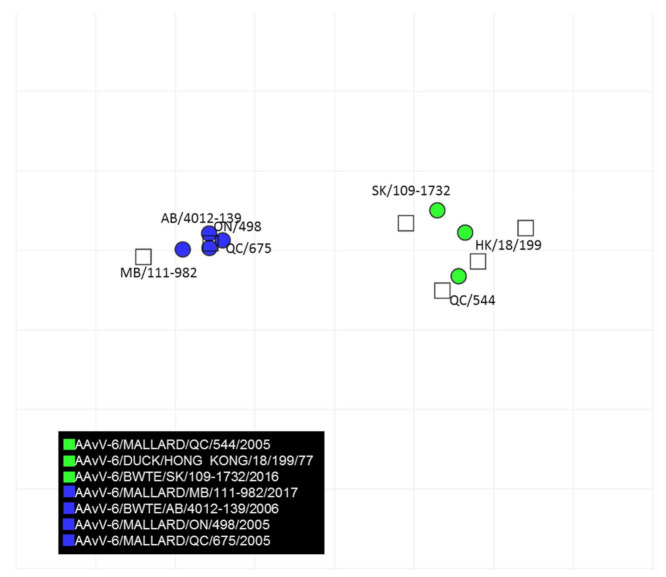
Antigenic map developed based on cross-hemagglutination inhibition test results of AAvV-6 viruses and antisera developed to each genotype of AAvV-6. The vertical and horizontal axes represent the antigenic distance and the spacing between each grid lines represents a distance of 1 antigenic-unit distance which corresponds to a 2-fold dilution in the HI assay.

The results of the cross-hemagglutination inhibition test are summarized in Table 2. There was minimal cross reactivity between viruses within genotype 2. The cross hemagglutination inhibition titer difference between viruses with in genotype 1a and 1b was minimal indicating they are antigenically close. The cross hemagglutination inhibition data was computed to generate an antigenic map quantifying the antigenic relationships between AAvV-6 strains (Figure 4). Based on antigenic cartography, AAvV-6 genotype 2 (blue) and genotype 1 groups (green circles) are antigenically distinct. The antigenic relatedness within AAvV-6 genotype 2 viruses were close. In contrast, AAvV-6 genotype 1 group viruses showed antigenic heterogeneity indicating existence of possible antigenic variants.

## 4. Discussion

The current study demonstrates for the first time, the presence of both genotype 1 and 2 AAvV-6 viruses in wild waterfowl in North America. The AAvV-6 viruses were isolated from wild waterfowl captured from four North American wild waterfowl flyways—the Atlantic, Mississippi, Central, and Pacific over a period of more than 10 years. In Genbank there currently exist only 11 full genome sequences for AAvV-6 viruses, all isolated from Eurasia. The 16 Canadian isolates from this study greatly enhance the number of full genome sequences reported. Fourteen of the sixteen viruses reported in this study are of genotype 2, a heretofore under represented genotype when it comes to full genome sequence in the Genbank database.

Genomes of AAvV-6 are dependent upon the genome length being a multiple of six for efficient genome replication [10]. Consistent with this, BWTE/SK/109-1732/2016 and mallard/QC/544/2005 have genome lengths of 16,236 nt. The remaining AAvV-6 viruses isolated were all of 16,230 nt and feature the same six nucleotide deletion upstream of the fusion gene as occurs in the IT4524-2 virus [9]. The fusion gene cleavage site likewise differentiates the viruses into two groups (Table 1) along lines similar to the serology. For Newcastle disease viruses, a dibasic fusion gene cleavage site accompanied with an F residue at the N-terminus of the F1 protein is often used to determine virulence of the virus [27]. None of the viruses isolated contain a dibasic motif accompanied with an F residue that would indicate velogenic virulence. Chickens were inoculated with a virus from each group. The chickens exhibited no clinical signs and appeared healthy throughout the course of the infection, with no deaths or illness occurring. These results are consistent with the previous findings of Chen et al. [14] whose mean death time score was >168 h and intracerebral pathogenicity index (ICPI) was 0, indicating the virus to be avirulent.

The genetic characterization of the AAvV-6 viruses studied determined the viruses to have a typical AAvV-6 genome structure, encoding seven structural proteins in the order 3′-leader-N-P-M-F-SH-HN-L-trailer-5′. Based upon sequence analysis and ORFs, predicted co-transcriptional modifications of the P gene through RNA editing produce putative V proteins of genotype 2 viruses that were strongly similar throughout their alignment. The genotype 2 viruses isolated from the Central North American wild waterfowl flyway from the province of Manitoba in 2006 have a distinct, shorter C-terminus than those from 2005 and 2017. This difference might be a result of genetic diversification of viruses circulating in different geographic areas or at different times. The V protein C-termini of the strains mallard/AB/4012-139/2006 and mallard/AB/4012-150/2006 have an identical sequence to that of duck/Italy/4524-2/2007 [26], whilst the rest of the Canadian genotype 2 viruses have a C-termini sequence extended by an additional 7 amino acids, that are nearly identical to one another. The sequence differences amongst the various W and V proteins reflect the patterns seen in the phylogenetic trees.

Analysis was conducted through the generation of phylogenetic trees of the full genome and the fusion gene, which to date has been one of the primary methods of genotyping AAvV-6 viruses. Along with the viruses in this study, additional viruses obtained from Genbank were used to generate Maximum Likelihood trees. Surprisingly, the two Canadian genotype 1 isolates do not cluster closest to one another. Rather, mallard/QC/544/2005 clusters well with existing AAvV-6 viruses irrespective of geographic location and year of isolation. The isolate BWTE/SK/109-1732/2016 is quite distinct and is the basal member of genotype 1 viruses. This unique clustering is attributable to differences located throughout the entirety of the coding and non-coding regions. The genotype 2 Canadian isolates cluster quite strongly together, with the two isolates obtained from Mallards in 2017 showing slight separation from the other genotype 2 isolates. In this instance, it is most likely that the differences in clustering observed are due to time of isolation rather than geography as the isolates from 2005 to 2006 years span the entirety of Canada, and were collected from each of Canada’s four migratory flyways. The 2017 isolates were obtained from birds sampled in central Canada, the Mississippi flyway, a flyway that in 2005 was represented by AAvV-6/mallard/ON/498/2005.

The viruses isolated in the current study hemagglutinated chicken red blood cells with the exception of AAvV-6/mallard/MB/111-986/2017. Although uncommon, a similarly non-hemagglutinating virus was isolated from a mallard in China [14]. Testing of the isolated AAvV-6 viruses against a panel of reference antisera developed against influenza A viruses (H1-16) and AAvV (1-9) showed no cross reactivity other than to AAvV-6.

Genetically, BWTE/SK/109-1732/2016 and mallard/QC/544/2005 grouped within a subgroup featuring the prototype virus (AAvV-6 duck/Hong Kong/18/199/77), whereas the vast majority of the other Canadian AAvV-6 viruses were in the second subgroup. When tested in HI assay with the reference antisera AAvV-6 duck/Hong Kong/18/199/77, the phylogenetic grouping is echoed antigenically. Genotype 2 viruses exhibited 8-fold or lower HI titers compared to the aforementioned genotype 1 viruses. The decrease in HI titers amongst non-heterologous subgroups had been previously observed by researchers examining Italian AAvV-6 viruses [9]. Convalescent anti-sera raised against the Italian strain IT4524-2 as well as the prototype virus were used to determine the HI titers of the Italian viruses. The homologous HI titer for IT4524-2 was 4-fold higher than the heterologous antiserum, leading researchers to conclude the existence of two antigenic subgroups within AAvV-6 [9].

For AAvV-6 viruses, there currently exists no genotype classification criteria [13,14]. In this absence, researchers have defaulted to classification criteria as established by Diel et al. [28] for AAvV-1, relying upon an evolutionary distance of greater than 10% to denote a new genotype. BWTE/SK/109-1732/2016 appears as a distinct branch on the fusion gene tree, and pairwise distances to the other genotype 1 viruses range from 16.0–19.6%. Unfortunately it is a lone isolate that may belong to an under-represented group that is genetically divergent from other genotype 1 viruses. However, in keeping with the Diel classification system [28], this cannot be resolved until at least four independent isolates are available. Recent estimates of inter-populational evolutionary distances between groups have been calculated at 47.6% [14]. The data set involved a total of 24 sequenes, 13 from genotype 1 and 11 from genotype 2. With the increase of the current dataset to 42 mucleotide sequences, the between group distances was expanded to 91.9%. The addition of AAvV-6/SK/109-1732/2016 greatly affected the inter-populational distance (supplement Table S2). Taken together, the serological and phylogenetic results exhibited by the two subgroups provide strong justification for the existence of two genotypes circulating in Canada’s wild birds.

A real-time RT-PCR assay was developed to detect and type AAvV-6 viruses based upon their respective genotypes. The vast majority of data available in Genbank is for the fusion gene. Sequence data of all known AAvV-6 fusion genes and those obtained in this study were aligned using MAFFT [22]. A roving 200 nucleotide window that contained conserved sites for primers and probes that could also serve to differentiate the two genotypes was scanned along the fusion gene alignment. Following in silico analysis, primers and hydrolysis probes were identified that resulted in a 191 bp amplicon. The limit of detection was determined to be 8.64 × 10^1^ and 1.69 × 10^2^ copies for genotypes 1 and 2, respectively. Swabs of experimentally infected chickens and viral isolates were tested for cross-reactivity, none was observed. Typical Ct values of the isolates were quite strong, ranging from 9~15. RNA from avian viruses commonly found in wild birds was used to examine the specificity of the assay. None of the Influenza A or avulaviruses tested were detected, indicating good specificity of the assay. All the viruses isolated in this paper were screened with the real-time RT-PCR assay and all were successfully identified with the correct genotype PCR probe based upon sequencing results. The developed duplex real-time RT-PCR assay was successfully used to study the shedding pattern of experimentally infected leghorn chickens with AAvV-6. Both strains replicated in chickens, causing subclinical infection and exhibited low levels of oral shedding. In another pathogenicity study performed by Hee et al. [10], 3-week old ducks were infected intranasally with AAvV-6 and the virus was recovered from tracheal and lung tissue samples, but not from the spleen or brain tissues, reflecting the shedding pattern prevalence seen in our PCR study. In chickens, viral replication was mostly restricted to the trachea and lungs of the infected birds.

In conclusion, our current study demonstrates the existence of AAvV-6 in North American wild waterfowl sampled in all four North American wild bird flyways. The Canadian AAvVs-6 are phylogenetically similar to Eurasian strains. These viruses were serendipitously and sporadically isolated from samples that were also positive for avian influenza virus over the span of more than 10 years. Our novel RT-PCR assay can be used to conduct larger scale surveillance for AAvV-6 in swabs collected from wild waterfowl, potentially in conjunction with AIV testing, in order to assess the apparent prevalence, distribution, and implications of AAvV-6 to waterfowl health and ecology, and to Canada’s poultry industry.

## Figures and Tables

**Table 1:** AAVV-6 fusion real-time RT-PCR primers and probes.

Primer and Probe	Genotype	Position
Forward 1 (Genotype-1)	3′-CACCCTTAAYCGAATTTTTACACC-5′	252–275
Forward 2 (Genotype-2)	3′-CACATTGAACCGCATATTCACRCC-5′	252–275
Rev. 1–2 (Genotype type-1 & 2)	3′-GCYCTTAACCARGCCCAGGA-5′	442–443
Probe (Genotype-1)	CALFluorOrange560-CAACCAGAACCCTGCTCCAG-BHQ1	327–346
Probe (Genotype-2)	FAM-CTCACCTCACTCCATACGTG-BHQ1	

**Table 2:** Cross hemagglutination inhibition assay test results AAvV-6 strains isolated in Canada from 2005–2016. Same colors indicate homologous titers for each virus and the corresponding antiserum.

Virus	Genotype	Antisera					
		Duck/HongKong/18/199/77		BWTE/SK/109-1732/2016		Mallard/QC/675/2005	
		Ck#1	Ck#2	Ck#483	Ck#485	Ck#602	Ck#604
Mallard/QC/544/2005	1	512	512	32	16	8	16
Duck/Hong Kong/18/199/77	1	512	1024	64	16	8	16
BWTE/SK/109-1732/2016	1	256	512	64	32	8	16
Mallard/MB/111-982/2017	2	128	64	4	4	64	128
BWTE/AB/4012-139/2006	2	128	32	4	4	128	128
Mallard/ON/498/2005	2	64	64	8	4	64	128
Mallard/QC/675/2005	2	128	64	8	4	64	128

**Table 3:** Characterization of Canadian AAvV-6 viruses. HI titres were derived against reference anti-sera from the prototype virus AAvV-6 duck/Hong Kong/18/199/77. ^a^ virus is missing the 14 nucleotides of the genome, ^b^ represents viruses in which only the fusion gene has been sequenced.

Virus	Length	Location (Province)	Year	Genotype	Cleavage Site	HA Titre	HI Titre	Genbank Accession Number
AAvV-6 mallard/QC/544/2005	16,236	Quebec	2005	G1	PAPEPR*LVGA	4096	128	MW338846
AAvV-6 BWTE/SK/OTH109-1732/2016	16,236	Saskatchewan	2016	G1	PAPEPR*LVGA	1024	64	MW338847
AAvV-6 mallard/AB/4012-139/2006	16,230	Alberta	2006	G2	SIREPR*LIGA	256	8	MW338858
AAvV-6 mallard/AB/4012-150/2006	16,230	Alberta	2006	G2	SIREPR*LIGA	64	16	MW338859
AAvV-6 mallard/QC/232/2005	16,230	Quebec	2005	G2	SIREPR*LIGA	256	4	MW338849
AAvV-6 mallard/QC/610/2005	16,216 ^a^	Quebec	2005	G2	SIREPR*LIGA	256	4	MW338850
AAvV-6 mallard/QC/675/2005	16,230	Quebec	2005	G2	SIREPR*LIGA	1024	8	MW338851
AAvV-6 mallard/QC/699/2005	16,230	Quebec	2005	G2	SIREPR*LIGA	512	16	MW338852
AAvV-6 mallard/QC/708/2005	16,230	Quebec	2005	G2	SIREPR*LIGA	512	8	MW338853
AAvV-6 mallard/QC/713/2005	1638 ^b^	Quebec	2005	G2	REPR*LIGA	2048	8	MW338862
AAvV-6 mallard/QC/743/2005	16,230	Quebec	2005	G2	SIREPR*LIGA	2048	4	MW338854
AAvV-6 mallard/BC/698/2005	16,230	British Colombia	2005	G2	SIREPR*LIGA	32	4	MW338856
AAvV-6 mallard/BC/704/2005	16,230	British Colombia	2005	G2	SIREPR*LIGA	256	4	MW338857
AAvV-6 American wigeon/NS/654/2005	16,230	Nova Scotia	2005	G2	SIREPR*LIGA	512	16	MW338848
AAvV-6 American wigeon/NS/664/2005	1638 ^b^	Nova Scotia	2005	G2	SIREPR*LIGA	128	16	MW338863
AAVV-6 mallard/ON/498/2005	16,230	Ontario	2005	G2	SIREPR*LIGA	64	8	MW338855
AAvV-6 mallard/MB/OTH111-982/2017	16,230	Manitoba	2017	G2	SIREPR*LIGA	512	8	MW338860
AAvV-6 mallard/MB/OTH111-986/2017	16,230	Manitoba	2017	G2	SIREPR*LIGA	did not hemaggluttinate		MW338861

## Data Availability

The data presented in this study are available in the article and Supplementary Materials.

## Supplementary Materials

The following are available online at https://www.mdpi.com/1999-4915/13/4/543/s1, Table S1: Estimates of evolutionary distances between AAvV-6 genotypes, Table S2: Pairwise estimates of evolutionary distances, File S1: W protein sequences, File S2: V protein sequences.
