# Supplementary material for: Genetic and Antigenic Characterization of Avian Avulavirus Type 6 (AAvV-6) Circulating in Canadian Wild Birds (2005–2017)
Source: Viruses. 2021 Mar 24;13(4):543. doi: 10.3390/v13040543 (PMC8064105; doi:10.3390/v13040543)
Supplement: Supplementary file 1 [file viruses-13-00543-s001.zip › Table S1.docx]

Table S1: Estimates of Evolutionary Distances between AAvV-6 genotypes inferred from the complete nucleotide Fusion gene sequences

|  | No. of base substitutions per site or standard error estimate | |
| --- | --- | --- |
| Genotype | 1 | 2 |
| 1 |  | (0.337) |
| 2 | 0.919 |  |

Table S1. Estimates of Evolutionary Divergence over Sequence Pairs between Groups

The number of base substitutions per site from averaging over all sequence pairs between groups are shown. Standard error estimate(s) are shown above the diagonal and were obtained by a bootstrap procedure (1000 replicates). Analyses were conducted using the Maximum Composite Likelihood model. The rate variation among sites was modeled with a gamma distribution (shape parameter = 1). The analysis involved 42 nucleotide sequences (genotype 1, n=15; genotype 2, n=27). Codon positions included were 1st+2nd+3rd+Noncoding. All positions containing gaps and missing data were eliminated. There were a total of 1637 positions in the final dataset. Evolutionary analyses were conducted in MEGA X.
